# A pilot study to show that asymptomatic sexually transmitted infections alter the foreskin epithelial proteome

**DOI:** 10.3389/fmicb.2022.928317

**Published:** 2022-10-17

**Authors:** Nyaradzo T. L. Chigorimbo-Murefu, Matthys Potgieter, Sonwabile Dzanibe, Zikhona Gabazana, Gershom Buri, Aditya Chawla, Bokani Nleya, Abraham J. Olivier, Rushil Harryparsad, Bridget Calder, Shaun Garnett, Lungile Maziya, David A. Lewis, Heather Jaspan, Doug Wilson, Jo-Ann S. Passmore, Nicola Mulder, Jonathan Blackburn, Linda-Gail Bekker, Clive M. Gray

**Affiliations:** ^1^Divisions of Medical Virology, Institute of Infectious Diseases and Molecular Medicine, University of Cape Town, Cape Town, South Africa; ^2^Division of Computational Biology, Institute of Infectious Diseases and Molecular Medicine, University of Cape Town, Cape Town, South Africa; ^3^Division of Chemical and Systems Biology, Institute of Infectious Diseases and Molecular Medicine, University of Cape Town, Cape Town, South Africa; ^4^Division of Immunology, Institute of Infectious Diseases and Molecular Medicine, University of Cape Town, Cape Town, South Africa; ^5^Department of Medicine, Edendale Hospital, Pietermaritzburg, South Africa; ^6^Western Sydney Sexual Health Centre, Western Sydney Local Health District, Parramatta, NSW, Australia; ^7^Westmead Clinical School and Sydney Institute for Infectious Diseases, University of Sydney, Sydney, NSW, Australia; ^8^Seattle Children’s Research Institute, Seattle, WA, United States; ^9^Department of Global Health, University of Washington, Seattle, WA, United States; ^10^Desmond Tutu HIV Centre, Cape Town, South Africa; ^11^Division of Molecular Biology and Human Genetics, Stellenbosch University, Cape Town, South Africa

**Keywords:** asymptomatic sexually transmitted infection, foreskin, foreskin proteome, HIV susceptibility, gene ontology enrichment analysis

## Abstract

There is limited data on the role of asymptomatic STIs (aSTIs) on the risk of human immunodeficiency virus (HIV) acquisition in the male genital tract (MGT). The impact of foreskin removal on lowering HIV acquisition is well described, but molecular events leading to HIV acquisition are unclear. Here, in this pilot study, we show that asymptomatic urethral infection with *Chlamydia trachomatis* (CT) significantly impacts the foreskin proteome composition. We developed and optimized a shotgun liquid chromatography coupled tandem mass spectrometry (MS)-based proteomics approach and utilized this on foreskins collected at medical male circumcision (MMC) from 16 aSTI^+^ men and 10 age-matched STI- controls. We used a novel bioinformatic metaproteomic pipeline to detect differentially expressed (DE) proteins. Gene enrichment ontology analysis revealed proteins associated with inflammatory and immune activation function in both inner and outer foreskin from men with an aSTI. Neutrophil activation/degranulation and viral-evasion proteins were significantly enriched in foreskins from men with aSTI, whereas homotypic cell–cell adhesion proteins were enriched in foreskin tissue from men without an aSTI. Collectively, our data show that asymptomatic urethral sexually transmitted infections result in profound alterations in epithelial tissue that are associated with depletion of barrier integrity and immune activation.

## Introduction

Globally, up to 75% of the human immunodeficiency virus (HIV)-infected men acquire HIV across the penis (Hladik and McElrath, 2008). Whilst HIV acquisition leading to infection has been extensively studied in the female genital tract (FGT), much less is known about the viral acquisition events in the male genitalia. Understanding the biological factors that increase the risk of acquiring HIV infection in the male genital tract (MGT) is important, so that alternatives to medical male circumcision (MMC) can be offered. The entry of infectious agents, such as HIV into the MGT, first involves penetration of the epithelial barrier, making up part of the shaft, glans, urethra, or foreskin, and consequent infection of host target cells. Various studies have shown that the surface area of the foreskin is directly related to the risk of HIV acquisition, (Kigozi et al., 2009) and its complete removal after MMC results in 54–61% risk reduction in HIV acquisition (Auvert et al., 2005; Bailey et al., 2007; Gray et al., 2007). Despite these findings, the biological mechanisms underlying reduced HIV acquisition after MMC are unclear. Several factors have been shown to play a role, including that the foreskin harbors HIV target cells (Hirbod et al., 2010), with reports of the inner foreskin containing a higher density of HIV target cells compared to the outer foreskin (Ganor et al., 2010; Lemos et al., 2014), and an inflammatory anaerobic microbiome which is reduced during MMC (Liu et al., 2013). The inner foreskin, being the foreskin compartment that is adjacent and covers the glans in uncircumcised men, has been reported to have a thinner stratum corneum (keratin layer) than the outer and an increased density of HIV target cells (Lemos et al., 2014), although this is controversial (Glynn et al., 2009; Dinh et al., 2010; Ganor et al., 2010). The ability to measure molecular differences in the foreskin compartments in individuals at risk for HIV acquisition would provide insight into potential mechanisms of HIV risk reduction afforded by MMC.

Symptomatic sexually transmitted infections (STIs), such as syphilis and Herpes simplex virus type 2 (HSV-2), are known to increase the risk of HIV acquisition and transmission due to the high levels of inflammation and the formation of lesions in men and women (Stamm et al., 1988; Boily and Anderson, 1996; Anzala et al., 2000; Glynn et al., 2009). *Chlamydia trachomatis* (CT) and *Neisseria gonorrhoeae* (NG), which are common asymptomatic STIs (aSTIs), can cause other reproductive health problems (Kohl et al., 2003; LeFevre and U.S Preventive Services Task Force, 2014). In the FGT, aSTIs have been shown to increase local inflammation (Bogaerts et al., 1999; Wiesenfeld et al., 2002), which appears to increase the risk of HIV acquisition (Mlisana et al., 2012; Masson et al., 2016). Inflammation has been shown to be associated with the influx of HIV target cells into the FGT of HIV-uninfected women (Arnold et al., 2016), and CD4 lymphocytes have been shown to be increased in the FGT of women with aSTI (Levine et al., 1998). Supportive evidence from *in vitro* data has shown that CT enhances HIV replication in co-culture (Ho et al., 1995; Buckner et al., 2016). We have also previously shown that CD4 + T cells, being HIV target cells, can migrate into the inner foreskin in response to inflammatory chemokines (Gray et al., 2019). However, there is a paucity of direct evidence regarding the effect of aSTIs on the molecular events in the foreskin that might predispose this tissue to become vulnerable to HIV infection. The impact of these urethral infections on the foreskin in the MGT is largely unknown. Here, we developed a novel metaproteome approach to analyze foreskin tissue and applied it to foreskins collected from adolescents undergoing MMC in a region of South Africa with high HIV prevalence (Ramjee et al., 2019). The aim was to understand how asymptomatic urethral STIs alter the foreskin proteome to create an environment conducive to HIV acquisition and replication. We hypothesized that proteins associated with barrier integrity and immune quiescence are disrupted upon a sub-clinical sexually transmitted infection (other than HIV). To test this, we interrogated the effect of an *in vivo* acquired asymptomatic STI on the inner and outer foreskin proteome using MS -based proteomics and compared the protein profiles with foreskins from age-matched men who were negative for the tested STIs. This approach allowed us to identify in an unbiased quantitative manner aSTI -associated alterations in proteomic profiles and protein interactions.

## Materials and methods

### Study cohort and detection of asymptomatic STIs

In this study, foreskin tissue was collected after MMC from men living in a region with high HIV prevalence in Kwa-Zulu Natal, South Africa, which we have previously reported (Gray et al., 2019). A full description of tissue procurement, laboratory processing, and freezing is detailed in the study (Gray et al., 2019). For the purposes of this study, fresh tissue was separated into inner and outer foreskin, and then portions were dissected into 0.5 cm × 0.5 cm pieces with a scalpel. These tissue samples were stored immediately after sample processing in vials and stored in liquid nitrogen until further use. Sixteen men (age 15–24 years) with aSTIs (eight with CT only infection or CT and/or other aSTI, detailed in Table 1) were age-matched to 10 control samples without any detectable aSTI as previously described (Gray et al., 2019) and was done retrospectively from the stored tissue archive. Men with symptomatic STIs were excluded from the study, and the presence of aSTI in urine samples was detected using real-time multiplex PCR (M-PCR) assays that qualitatively detect *Neisseria gonorrhoeae* (NG), *Mycoplasma genitalium* (MG), *Trichomonas vaginalis* (TV), CT, and herpes simplex virus types 1 and 2 (HSV 1 and 2).

**TABLE 1:** Description of the number of samples used for proteomic analysis.

	Control	aSTI group
Number of individuals	10	16
Age	15–24 years	15–24 years
Sex	Male	Male
aSTI	None detected	*Chlamydia Trachomatis only* (8) *Chlamydia Trachomatis* and *Mycoplasma genitalium* (3), *Herpes simplex virus 2* (1), *Neisseria gonorrhoeae* (2), *Chlamydia Trachomatis, Neisseria gonorrhoeae* and *Mycoplasma genitalium* (1), *Trichomonas vaginalis* (1)

Thirty-two aSTI + samples (16 inner and 14 outer foreskin samples) and corresponding age-matched 20 STI samples (10 inner and 10 outer foreskin samples).

### Tissue lysis, protein extraction, and peptide generation

The tissue was thawed on ice in 4 volumes of extraction buffer [4M Guanidine HCl, 100 mM NaCl, 5 mM TCEP (tris-carboxyethyl phosphine), 2 mM EDTA, 1% OGP (octylglucopyranoside) in 100 mM TEAB (triythelammonium bicarbonate)], together with glass beads. Once thawed, the samples were vortexed for 1 min before being placed back on ice. The sample was sonicated three times for 30 s, centrifuged for 10 min at 12,000 × g, and the supernatant was removed and stored on ice. This process was repeated three times, and the total protein was precipitated from the pooled supernatants with ice-cold acetone overnight at –20^°^C.

Different tissue disruption and protein solubilization techniques were investigated. Sonication to aid tissue disruption in RIPA buffer (high SDS) was performed. Protein quantities of the extracts were measured using the bicinchoninic acid (BCA) assay suitable for proteins in high SDS solutions. Two methods of peptide preparation for mass spectrometry (MS) were utilized, namely, filter-aided sample preparation (FASP), which is an on-column proteolysis method, and in-solution trypsin (IS) digestion. For the FASP protocol, 200 μg of protein was used and peptides were prepared for label-free quantitative mass spectrometry by trypsinization (Promega) on a filter as previously described in Nel et al. (2015). Briefly, protein extract was placed in an Ultracel 30 kDa molecular cut-off centrifugal unit (Amicon Ultra, Merck) and washed in 8 M urea buffer to remove the detergent. Disulfide bonds were reduced and alkylated in 0.05 mM iodoacetamide (IAA), and the excess IAA was washed off by 50 mM ammonium bicarbonate buffer. Trypsin proteolysis (1:50 sequencing grade trypsin to protein extract) (Promega) was performed at 37^°^C for 18 h in a humidified chamber.

For in-solution (IS) digestion, 200-μg protein pellets were dissolved in 100 mM TEAB containing 4 M guanidine-hydrochloride. Samples were reduced by adding 50 mM triscarboxyethyl phosphine (TCEP; Fluka) in 100 mM TEAB (final concentration 5 mM TCEP) and incubated for 30 min in a room at a temperature of 60^°^C. Following the reduction of cysteine, residues were modified to methylthiols using 200 mM methane methylthiosulfonate (MMTS; Sigma) in 100 mM TEAB (final concentration 20 mM) for 30 min. After modification, the samples were diluted to 98 μL with 100 mM TEAB. Proteins were digested by adding 5 μL of trypsin (New England Biolabs) solution (1 μg/ μL) and incubating for 18 h at 37^°^C. The samples were dried down and resuspended in 100 μL of 2% acetonitrile (Fluka): water and 0.1% formic acid (formic acid; Sigma). Residual digest reagents were removed using an in-house manufactured C_18_ stage tip (Empore Octadecyl C_18_ extraction discs; Supelco). The 20-μL sample was loaded onto the stage tip after activating the C_18_ membrane with 30 μL of methanol (Sigma) and equilibration with 30 μL of 2% acetonitrile: water and 0.05% TFA. The bound sample was washed with 30 μL of 2% acetonitrile: water and 0.1% FA before elution with 30 μL of 50% acetonitrile: water and 0.1% FA. The eluate was evaporated to dryness. The dried peptides were dissolved in 20 μL of 2% acetonitrile: water and 0.1% FA for LC-MS analysis.

### Gel-based separation and digestion

**FIGURE 1:**
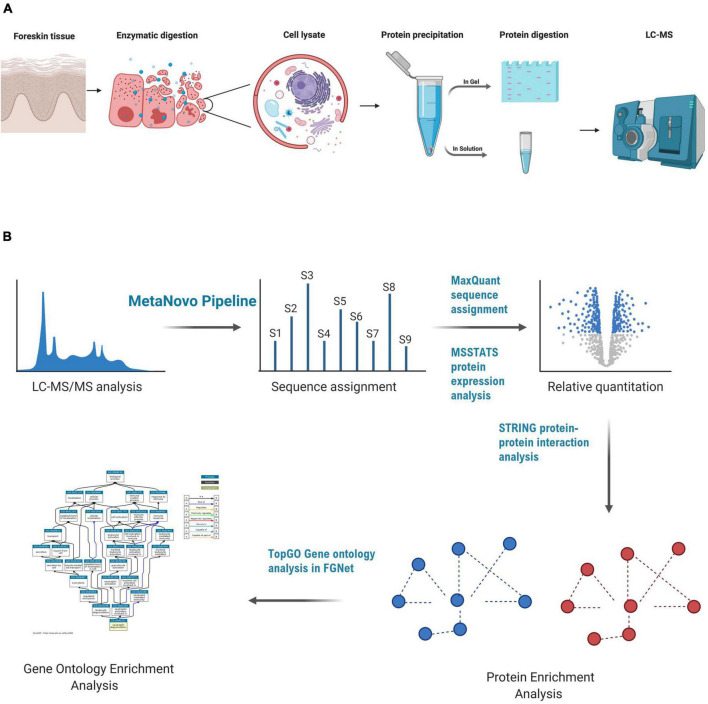
LC-MS-based proteomics of foreskin samples and data analysis methods used to assess the impact of aSTI on the foreskin proteome. **(A)** Equal mass of inner and outer foreskin samples were dissected, and proteins were solubilized and digested with trypsin enzyme to create peptides that were analyzed by LC-MS. In-gel digestion method, FASP, and in-solution digestion methods were performed to determine the appropriate method for foreskin peptide generation. **(B)** Data analysis method after the acquisition of peptide mass spectra composed of database optimization using the MetaNovo pipeline which was used to optimize sequence assignment. Subsequent label-free quantification of identified protein groups was performed using MaxQuant, and differential protein expression was analyzed using the MSSTATS protein analysis pipeline for relative quantification of proteins from aSTI foreskins compared to age-matched controls. GO enrichment analysis of differentially expressed proteins was conducted in FGNet using the TopGO function. This figure was generated using BioRender.com.

A total of 100 μg of protein lysate from an individual’s outer foreskin tissue lysate was separated using polyacrylamide gel electrophoresis using the Invitrogen NuPAGE mini gel system. The lysate was mixed with 2x sample loading buffer and distributed into seven wells (15 μg in each) of a gradient gel. Precast NuPAGE 4–12% Bis-Tris mini gels (1.0 mm × 12 wells) were used. Lysates were separated (400 V, about 40 min) with a NuPage 4–12% Bis-Tris gel 1.0 mm × 12 holes (Invitrogen). The gel was stained using Coomassie blue and five zones (A-E) were divided and processed into sections according to the in-gel trypsin protein digestion method described by Shevchenko et al. (2006). A summary of the methods followed for sample preparation is shown in Figure 1A.

### Liquid chromatography-mass spectrometry analysis

The peptides generated from the gel-based preparation and FASP methods were desalted in preparation for MS analysis on homemade columns made of Empore Octadecyl C18 solid-phase extraction disk (Supelco). Activation of the C18 column was performed by 80% acetonitrile and equilibrated by 2% acetonitrile. Desalting of peptides and subsequent elution were performed as above, after which peptides were dried under vacuum and resuspended in 2% ACN and 0.1% formic acid at 250 ng/μL. All the washes and rinses were performed in triplicate using HPLC-grade solvents. Peptide identification and quantification were then performed by ultra-high-performance liquid chromatography coupled in line with a Q Exactive hybrid quadrupole Orbitrap mass spectrometer (Thermo). Briefly, separation of the peptides was done on a laboratory packed 100 μM ID × 20 mm pre-column connected to a 75 μM, 5 μM × 500 mm 100 Å pore size C18 analytical column from Phenomenex. This was conducted on a Dionex Ultimate 3,500 RS nano UPLC. The flow rate used was 300 μL/min at a pressure of less than 350 bars. The mobile phase for loading 1 μg of desalted peptides was composed of 2% acetonitrile and 0.1% formic acid. A gradient was used to elute the peptides with 10 min of the previously mentioned mobile phase, then increased to 25% acetonitrile for 115 min, then to 35% acetonitrile for 5 min, and finally to 80% acetonitrile.

Mass spectra were acquired in a data-dependent manner, with automatic switching between MS and MS/MS scans using a “Top-10” method. MS1 spectra were acquired at a resolution of 70,000 with a target value of 3 × 10^6^ ions or a maximum integration time of 250 ms. The scan range was limited from 300 to 1,750 m/z. Peptide fragmentation was performed *via* higher energy collision dissociation (HCD) with the energy set at 25 NCE. The intensity threshold for ion selection was set at 1.7 × e^4^ with charge exclusion of z = 1 and z > 5. The MS/MS spectra were acquired at a resolution of 17,500, with a target value of 2 × 10^5^ ions or a maximum integration time of 120 ms, and the isolation window was set at 4.0 m/z.

The method of analysis for the peptides prepared by IS digestion was performed on a Thermo Scientific Ultimate 3,000 RSLC equipped with a 0.5 cm × 300 μm C_18_ trap column and a 35 cm × 75 μm in-house manufactured C18 analytical column (Aeris C18, 3.6 μm; Phenomenex). The solvent system employed for loading was as follows: 2% acetonitrile: water, 0.1% FA, Solvent A: 2% acetonitrile: water and 0.1% FA, and Solvent B: 100% acetonitrile: water. The samples were loaded onto the trap column using loading solvent at a flow rate of 15 μL/min from a temperature-controlled autosampler set at 7^°^C. Loading was performed for 5 min before the sample was eluted onto the analytical column. Flow rate was set to 400 nL/min and the gradient generated was as follows: 2.0 –10% B over 5 min; 5-25% B from 5 to 50 min using Chromeleon non-linear gradient 6, and 25–45% from 50 to 65 min. Chromatography was performed at 50^°^C, and the outflow was delivered to the mass spectrometer through a stainless-steel nano-bore emitter.

MS was performed using a Thermo Scientific Fusion mass spectrometer equipped with a Nanospray Flex ionization source with a stainless-steel emitter. Data were collected in positive mode with the spray voltage set to 1.9 kV and ion transfer capillary set to 275^°^C. Spectra were internally calibrated using polysiloxane ions at m/z = 445.12003 and 371.10024. MS1 scans were performed using the orbitrap detector set at 120,000 resolutions over the scan range 350–1,650 with AGC target at 3 × 10^5^ and a maximum injection time of 40 ms. Data were acquired in profile mode.

The MS2 acquisitions were performed using monoisotopic precursor selection for the ion with charges + 2 to + 6 with error tolerance set to ± 10 ppm. Precursor ions were excluded from fragmentation once for a period of 30 s. Precursor ions were selected for fragmentation in HCD mode using the quadrupole mass analyzer with stepped HCD energy set to 32% ± 8%. Fragment ions were detected in the orbitrap mass analyzer set to 50,000 resolution. The AGC target was set to 1 × 10^4^ and the maximum injection time to 120 ms. The data were acquired in centroid mode.

### Bioinformatic analysis

Maxquant software package version 1.5.0.3 with integrated Andromeda search engine was used to analyze the raw MS spectra (Cox and Mann, 2008) search against a UniProt human and microbial protein database (ca. 65 million entries; July 2017 release) filtered using the MetaNovo pipeline^1^ and concatenated with the human reference proteome to create a database of 146,885 entries, as previously described (Potgieter et al., 2019; Alisoltani et al., 2020). Briefly, the MS/MS database search was performed using default settings, with a 20 ppm mass tolerance for the main search. Cysteine carbamidomethylation was selected as a fixed modification. Trypsin was selected as the protease, with up to two missed cleavages allowed. Results were filtered by a 0.01 false discovery rate at both protein and peptide levels. The minimum length of acceptable identified peptides was set at seven amino acids. Log2-transformed label-free quantitation (LFQ) values were used to identify dysregulated human and microbial proteins significantly.

### Protein quantification and statistical analysis

Statistical testing and relative quantification of proteins were performed using MSstats in R (Choi et al., 2014). Protein significance analysis was carried out based on a linear mixed-effects model to calculate fold changes and *p*-values. Relative quantification of proteins was performed, and the differential abundance was defined using a Student *t*-test measuring differences in the log2 fold change of protein expression between aSTI participant samples and controls. *P*-values were adjusted among all the proteins in the specific comparison using the approach by Benjamini and Hochberg (adj. *p*-value), and a cut-off of 0.05 was applied.

### Hierarchical clustering and dimensional reduction

Multidimensional scaling (MDS) of protein expression and PERMANOVA were determined using the vegan R package (Dixon, 2003), and the unsupervised hierarchical clustering of protein levels identified from the foreskin samples was used to visualize variation in the relative protein quantification in each sample using QlucoreOmics Explorer, Sweden. FDR for protein expression was set at *q* = 0.05.

### Functional enrichment analysis and gene ontology analysis

For the gene ontology (GO) analysis, we utilized the TopGO package as part of the FGNet R framework (Alexa and Rahnenführer, 2020). The analysis was completed with the classic algorithm and a Fisher’s exact test, with a *p*-value threshold of 0.01. The logFC values and GO terms were exported and visualized using bubble plots with the GOplot R package. For the bubble plots, the GOplot packages separate the GO terms by biological processes (BP), molecular function (MF), and cellular compartment (CC). The size of the bubble represents the number of proteins in the GO, and the y-axis represents the negative log of the adjusted *p*-value. The z-score represents the difference in the number of proteins in the GO term with positive and negative logFC values, normalized to the total count. A summary of the bioinfomatic analysis and data analysis methods followed is shown in Figure 1B.

### Flow cytometric analysis of human immunodeficiency virus susceptible from foreskin epidermal sheets

The method used for isolating HIV target cells expressing CD45, CD4, and CCR5 has been described previously (Gray et al., 2019). We performed these experiments on fresh inner and outer foreskin tissue obtained from a different cohort in Cape Town HREC 568/2020 (University of Cape Town). Isolated cells were stained with the following antibodies against CD45 (BV785 clone HI30), CD3 (APC-Cy7 clone UCHT-1), CD4 (AF700 clone RPA-T4), and CCR5 (BV711 clone J418F1) from men with and without aSTI.

## Results

### Optimization of foreskin processing for shot-gun LC-MS/MS analysis

IS digestion in a Guanidine/TCEP-based buffer was chosen over the FASP or chloroform/methanol IS digestion protocols, as it yielded a better-quality peptide spectrum by LC-MS/MS (Supplementary Figure 1) with the least amount of background and better resolution of peptide peaks. We found that this approach yielded higher peptide concentrations from the pilot samples tested (*n* = 2) when compared to the others (Supplementary Figure 1, I–III).

### Distinct clustering of aSTI + and age-matched asymptomatic STIs control foreskin proteomes

**FIGURE 2:**
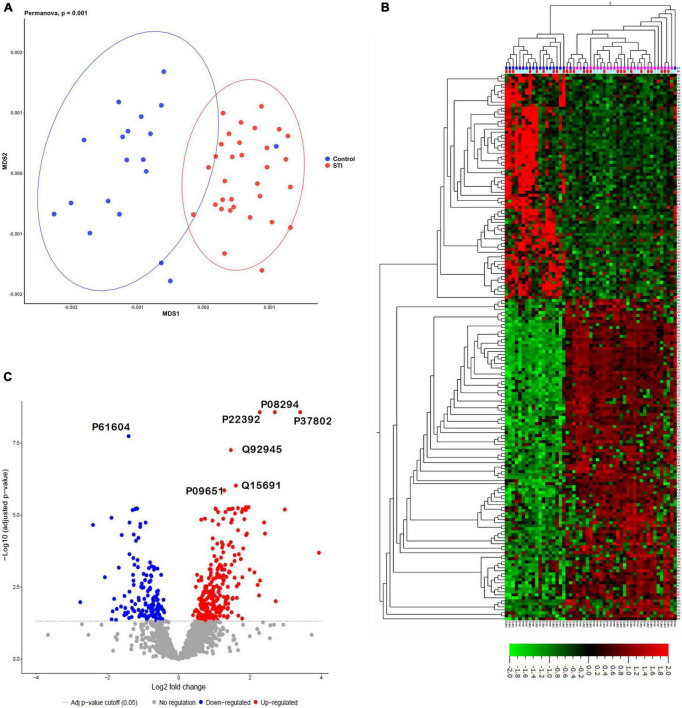
Distinct proteomes of inner and outer foreskins from men with aSTI compared to age-matched controls without aSTI. **(A)** MDS plot showing dissimilarity of proteomes in aSTI foreskins compared to controls, **(B)** volcano plot showing 400 significantly up- or downregulated proteins (adjusted *p* = 0.05) comparing differences in log_2_ fold changes between control (STI) and aSTI foreskin protein expression. Gray dots indicate non-significant protein fold changes, blue dots represent proteins with a negative log_2_ fold change (higher expression in STI samples), and red dots depict those with a positive log_2_ fold change (higher expression in aSTI samples). **(C)** Unsupervised hierarchical clustering showing differential protein expression of fold changes in the expression of the foreskin proteins in control samples and aSTI foreskins (*q* = 0.01).

We identified 11,497 non-redundant peptides from 294,793 assigned spectra (out of 2,386,055 spectra submitted, with a 12.4% identification rate). From these peptides, 2,108 protein groups were identified and quantified using label-free quantification in MaxQuant (Potgieter et al., 2019). Protein groups are protein identities that cannot be unambiguously identified by identified peptides in the mass spectrometer. Using MDS, the identified foreskin peptides clustered distinctly by STI status (PERMANOVA *p* = 0.001; Figure 2A). We next determined the relative quantification of proteins using MSstats R proteomics package and found that 400 of 2,108 protein groups were significantly differentially expressed (DE) between the STI + and STI- groups (Figure 2B; adj. *p* ≤0.05). Two hundred and sixty-three protein groups were upregulated in aSTI compared to no STI group, and 137 were downregulated (Supplementary Datasheet 1). The top 10 upregulated protein groups identified in no STI group included parathymosin (P20962), clathrin light chain B (P09497), 60S ribosomal protein L13a (P40429), a component of the gamma interferon-activated inhibitor of translation (GAIT), and an unreviewed bacterial glycosyl transferase family protein (M0GAG6). Upregulated proteins in the presence of an aSTI included a bacterial putative 2-succinyl-6-hydroxy-2,4-cyclohexadiene-1-carboxylate synthase (A0A094JCH3), protein S100-A11 (P31949), peroxiredoxin 4 (Q13162), extracellular superoxide dismutase (P08294), and ferritin (P02794). Proteins significantly enriched in the presence of an aSTI compared to no STI were visualized using unsupervised hierarchical clustering (Figure 2C; FDR *q* = 0.01). Protein group clustering, however, was not significantly different between inner and outer foreskin. Overall, these data show that the proteomic profile of foreskin tissue is affected by aSTIs with both inner and outer foreskin uniformly affected.

### Enriched molecular functions and biological processes in inner and outer foreskins from men infected with an aSTI

We next interrogated functional differences between foreskins derived from aSTI + and aSTI- males. Using GO enrichment analysis, we assigned holistic biological functions to all DE human proteins, as shown in Supplementary Datasheet 2. Supplementary Figure 2 shows the GOFacet bubble plot of TopGo (Alexa and Rahnenführer, 2020) enriched functions from the significantly differentiated proteins and were categorized as follows: (a) biological process, (b) CC, and (c) MF. The bubble plot depicts each GO as a bubble with the size of the bubble correlating to the number of proteins represented in each ontology. It can be seen that certain GOs with corresponding negative fold change (x-axis, z-score) were enriched in control proteomes and others with positive fold changes were enriched from aSTI + proteomes. A total of 433 GOs were identified and plotted in the figure with the most significant (Supplementary Figure 2) being annotated. The full list of GOs with the corresponding proteins and adjusted *p*-values are listed in Supplementary Sheet 2 (negative and positive FC tabs). This unbiased approach revealed a rich array of differentially enriched GOs, with some common between the groups, such as GO: 0005912: Adherens junctions, some being only significantly enriched in aSTI- proteomes (GO: 2001271: Negative regulation of cysteine-type endopeptidase activity involved in the execution phase of apoptosis), and some enriched in aSTI + proteomes (GO: 0043280: Positive regulation of cysteine-type endopeptidase activity involved in the apoptotic process).

**FIGURE 3:**
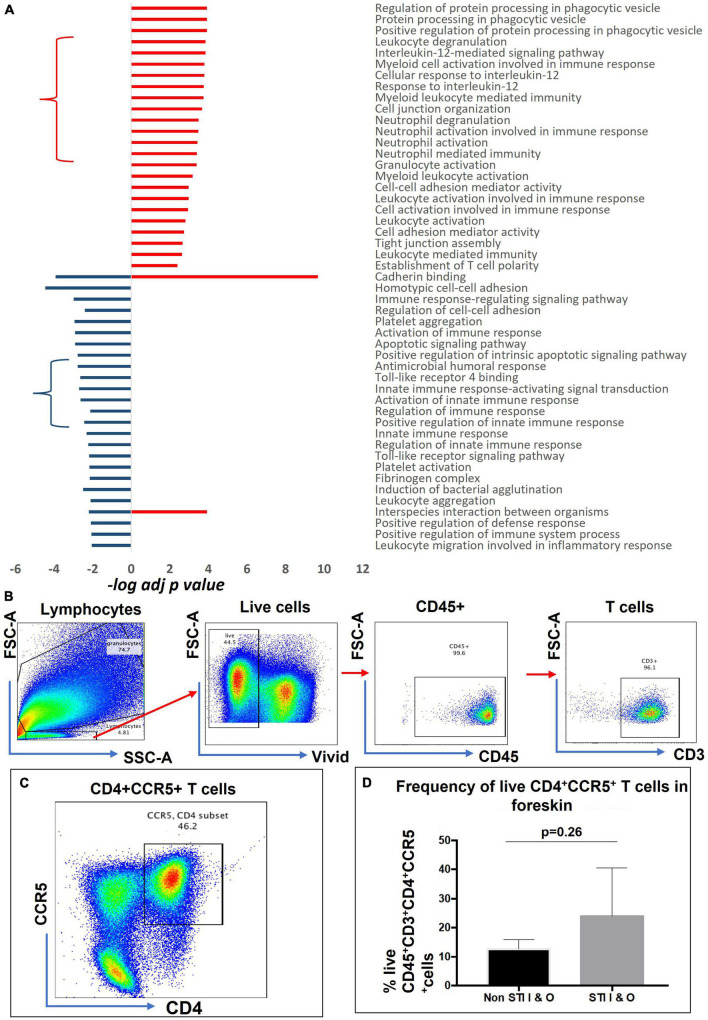
Distinct functions enriched from proteins upregulated in aSTI foreskins and age-matched controls. **(A)** Significance of immune-related GO term enrichment in aSTI + (red bars) and aSTI- (blue bars) foreskin proteomes. A classical enrichment analysis tested (Fischer’s test) the significance of enrichment of the GO terms in the respective proteomes and is expressed as logarithms. **(B)** Multiparameter flow cytometry gating strategy applied to phenotype-isolated primary inner and outer foreskin leukocytes expressing CD3. Live cells were selected which expressed CD45 and CD3 as shown. **(C)** A representative bivariate flow cytometry plot showing single- and double-positive CD4 + CCR5 + cells gated from CD45 + CD3 + T cells. The boxed gate shows the percentage of double-positive T cells. **(D)** Percentage of CD45 + CD3 + CD4 + CCR5 + cells from combined outer and inner foreskins in STI- controls and STI + men. Error bars represent the standard deviation from the mean. The cells were gated from the grandparent (CD45 + CD3 + cells).

We selected a total of 100 GO terms, with 63 enriched in aSTI+ foreskins and 55 in aSTI- foreskins, with 18 GO terms being common to both groups based on the top significant immune-related functions (Supplementary Datasheet 2, Selected GO tab). Figure 3A depicts 49 of the 100 GO terms that were related to direct immunological functions to show which immune pathway themes were up- and/or downregulated in the presence of any aSTI. The GO terms used are those provided by the GO Consortium.^2^ Granulocyte activation (adj. *p*-value = 4.0 × 10^–4^, –log10 = 3.39), establishment of T-cell polarity (*p* = 4.0 × 10^–3^, –log10 = 2.38), regulation of protein processing in phagocytotic vesicle (adj. *p*-value = 1.2 × 10^–4^, –log10 = 3.92) were all upregulated in foreskins obtained from aSTI+ males, as shown in Figure 3A. Conversely, several functions related to innate immunity, such as innate immune response-activating signal transduction and positive regulation of innate immune response, were upregulated in aSTI- control samples (adj. *p*-value = 2.0 × 10^–3^ and 4.0 × 10^–3^, respectively, –log10 = –2.70 and –2.43) (Figure 3A, blue curly arrow). Two functions, that is, interspecies interaction between organisms and cadherin binding, were enriched in foreskins obtained from both STI + and STI– men but were elicited by different proteins and to different extents (e.g., Interspecies interaction between organisms: adj. *p*-value of 1.0 × 10^–4^ in aSTI samples and 6.0 × 10^–3^ in control samples (Supplementary Datasheets 1, 2 and Figure 3B).

### Enrichment of gene ontologies associated with the recruitment of HIV target cells and inflammatory pathways from proteins enriched in the foreskin of men with asymptomatic STIs

Interleukin (IL)-12 secretion by myeloid cells causes differentiation of naïve T cells into HIV-1 susceptible Th1 cells (Bottomly, 1988; Hsieh et al., 1993). The GO functions are as follows: cellular responses to IL-12 (GO: 0071349, adj. *p*-value = 1.6 × 10^–4^), IL-12-mediated signaling pathway (GO: 0035722, adj. *p*-value = 1.4 × 10^–4^) (Figure 3A in red curly arrow), myeloid leukocyte-mediated immunity (GO: 0002444, adj. *p*-value = 1.8 × 10^–4^), myeloid cell activation involved in immune response (GO: 0002275 adj. *p*-value = 1.6 × 10^–4^) amongst others (Figure 3A) were enriched in the proteomes of men with an aSTI. We validated the findings by investigating the presence of these HIV-susceptible cells (CD45+ CD3+ CD4+ CCR5+ cells, Figures 3B–D) in the inner and outer foreskins of new recruits with and without an aSTI. The frequency of these HIV target cells was markedly higher in men with an aSTI (Figure 3D), as previously shown by us and others (Masson et al., 2016, 2019; Gray et al., 2019).

**FIGURE 4:**
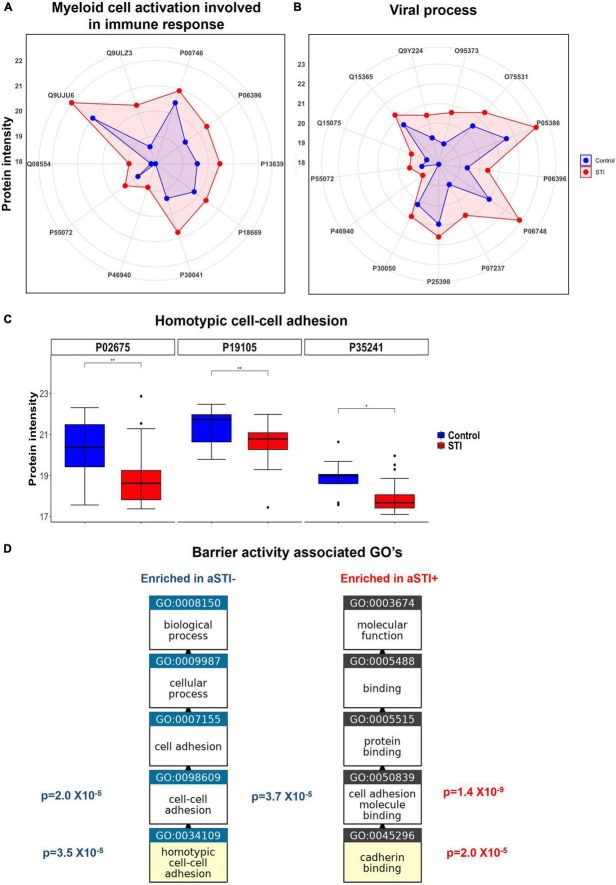
Enriched immunological functions showing the impact of aSTI on protein functions enriched in aSTI proteomes compared to age-matched controls. **(A)** Radar plot showing the intensity of proteins in aSTI + foreskins (red area) and control foreskins (aSTI-) (blue) with related myeloid cell activation functions. Protein names are depicted by UNIPROT numbers for ease of annotation. **(B)** Radar plot showing protein intensity of proteins enriched in GOs related to viral immune response. **(C)** Box and whisker plots showing expression intensities of protein eliciting homotypic cell-cell adhesion protein function and their names. **(D)** Enriched GOs with barrier integrity function enriched in foreskin samples aSTI + (red) and aSTI-(blue) with their corresponding adjusted *p*-values of enrichment shown. GO flow diagram from https://www.ebi.ac.uk/QuickGO/annotations.

Protein intensities of peroxiredoxin-6 (PRDX6, P30041), PYCARD (Q9ULZ3), and both ferritin light and heavy chains (FTL and FTH) were higher in aSTI foreskin compared to the control foreskin (red area compared to the blue area in the radar plot Figure 4A). These proteins interact with one another *in vivo* and are responsible for the enrichment of the biological process of neutrophil degranulation (GO: 0043312 adj. *p*-value = 3.7 × 10^–4^, Figure 3B). Neutrophil degranulation is a part of myeloid leukocyte activation a GO observed here to also be enriched in aSTI (GO: 0002274 adj. *p*-value = 6.5 × 10^–4^). PYCARD is a key component of one of the NLPS inflammasomes and is also involved in macrophage pyroptosis and inflammation (Agostini et al., 2004; Fernandes-Alnemri et al., 2007). S100A8, which plays a critical role in inflammation (Wang et al., 2018), was also significantly more expressed (adj. *p*-value of 5.0 × 10^–3^) at > 2-fold higher in aSTI foreskin compared to control samples (Supplementary Datasheet 1). The increased expression of proteins involved in myeloid activation in the foreskin of men with aSTI suggests an increased proinflammatory state within the foreskin.

### Significantly higher expression of proteins involved in host–viral interaction in proteomes from men with asymptomatic STIs

We observed significantly higher expression of cellular proteins that are known to support viral immune acquisition in aSTI+ foreskin compared to men without aSTI. These include host cell co-factor proteins that facilitate HIV-1 infection, such as BANF1 (O75531), importin (O95373), NPM1 (P06748), SNW domain-containing protein 1 (Q13573), and PCBP1 (Q15365). Their expression in different foreskin samples is shown in Figure 4B. Barrier to auto-integration factor BANF1 (O75531 in Figure 4B) is a host protein that is appropriated by retroviruses, including HIV-1, to promote viral DNA integration into the host genome (Harris and Engelman, 2000). Other proteins involved in HIV-1 replication were also elevated in aSTI foreskins; Importin IPO7 has been shown *in vitro* to mediate the nuclear import of HIV-1 reverse transcription complex integrase and HIV-1 rev during infection (Szklarczyk et al., 2019). NPM1 (P06748 in Figure 4B) is also an important protein in the nucleolar localization of the HIV-1 protein Tat (Li, 1997). In addition to this, SNW domain-containing protein 1 (Q13573 in Supplementary Datasheet 1) is also recruited by HIV-1 Tat and is involved in Tat transcription (Brès et al., 2005). Another co-factor used by HIV that is DE in the foreskin of men with aSTI is PCBP1, the Y-box binding protein 1 which supports early and late steps in HIV replication in the host, and was also significantly higher in men with aSTI (Weydert et al., 2018).

The protein SKP1 (P63208), which was also higher in aSTI proteome, is an important factor in the degradation of CD4 promoted by HIV-1 Vpu. It is recruited in the CD4 ubiquitination and subsequent proteasomal degradation of the receptor (Li, 1997). This interaction positively modulates HIV-1 infection (Levesque et al., 2003). It is noteworthy that GO: 0046596, which regulates viral entry into the host cell, was enriched in the aSTI proteomes [adj. *p* = 0.008 (Supplementary Datasheet 2)].

Furthermore, the foreskin proteomic profile of men with aSTI was enriched in proteins involved in suppressing innate viral response mechanisms, such as valosin-containing protein (VCP)/P55072 (Figure 4C). Together with its co-factor NPL4-UDF1, it reduces antiviral innate immune responses by suppressing antiviral signaling *via* degradation of RIG-I. VCP also suppresses the production of the innate antiviral type 1 interferon through its interaction with RIG-I (Hao et al., 2015).

### Ontologies related to barrier function enriched in control proteomes

Multiple ontologies related to maintenance of tissue barrier function were significantly enriched in foreskin proteomes of men without detectable STIs compared to men with aSTI. The biological process homotypic cell adhesion GO: 0034109 the attachment of a cell to a second cell of the identical type *via* adhesion molecules (QuickGO, 2022) was enriched (adj. *p*-value 3.5 × 10^–5^) in the proteomes of men without any detectable aSTI (shown in Figure 3B highlighted with a blue star). The proteins radixin (P35241), myosin regulatory light chain (J3QRS3), and fibrinogen beta chain were the proteins with higher expression in the aSTI- foreskin responsible for the enrichment and whose expression is shown in Figure 4C. Cell to cell adhesion (GO: 0098609) was also enriched in control proteomes (adj. *p*-value 2.0 × 10^–5^). Cadherin binding GO: 0045296 was significantly enriched in aSTI proteomes (Figure 3B). There was significant enrichment of cell adhesion molecules (GO: 0050839: Cell adhesion molecule binding) in aSTI+ proteomes (Supplementary Datasheet 2), such as cell–substrate junction proteins (GO: 0030055, Adherens GO: 0005912, and Anchoring junctions GO: 0070161). Altered barrier function during aSTI allows the influx of cells, such as dendritic or other immune cells into tissue (McEver and Zhu, 2010). Figure 4D shows the relatedness of the barrier function of GO terms enriched in the different proteomes.

### Ontologies related to humoral immunity enriched in control proteomes

The MF of Toll-like receptor 4 binding (GO: 0035662, Figure 3A and Supplementary Datasheet 2) was enriched in control proteomes (adjusted *p*-value 2.3 × 10^–3^), as well as the BP related to the Toll-like receptor signaling pathway (GO: 0002224, adjusted *p*-value 6.6 × 10^–3^). This term is a subclass of innate immune response-activating signal transduction (GO: 0002758, adjusted *p*-value 2.0 × 10^–3^), a subclass of GO: 0002218, Activation of innate immune response (adjusted *p*-value 2,4 × 10^–3^) also a subclass of GO: 0045089 Positive regulation of innate immune response (adjusted *p*-value 3,7 × 10^–3^), which is involved in the regulation of innate immune response (GO: 0045088, adjusted *p*-value 6.1 × 10^–3^). All of these are part of the innate immune response (GO: 0045087, adjusted *p*-value 4.9 × 10^–3^) related to the activation of immune response GO: 0002253 (*p*-value 1.2 × 10^–3^). All of these are shown in Figure 3A and listed in Supplementary Datasheet 2 Negative FC and Selected GO tabs. These contrasted with the cellular-based immune activation seen to be enriched in aSTI proteomes described above. In addition to this innate immunity-related GO enrichment in control proteomes, components of the humoral immune system, antimicrobial humoral response (GO: 0019730 adj. *p*-value 1.7 × 10^–3^), and induction of bacterial agglutination (GO: 0043152 adj. *p*-value = 3.2 × 10^–3^) were also enriched in control proteomes.

## Discussion

To our knowledge, foreskin proteomics has not yet been successfully performed. We developed a novel proteomic workflow for the analysis of foreskin tissue. LC-MS-based proteomics, which harnesses the analytical power of MS to analyze whole protein contents of biological systems, is a popular tool for investigating host–pathogen effects and interactions in disease settings (Hanash, 2003). The bioinformatic capabilities of analyzing proteomics data specifically in functional enrichment analysis can provide biological insight into perturbations to host systems caused by diseases. This information can be used in designing clinical tools for the diagnosis or discovery of therapeutic agents against pathogen effects. We show here an optimized method for the LC/MS-based proteomic analysis, including sample processing methods for analyzing clinical skin samples which are notoriously difficult. We were able to apply this to differentiate clinical samples and to provide biological insights into the effects of aSTIs in men living in a high HIV prevalence area of South Africa.

Symptomatic and aSTIs are known to induce an acute inflammatory response in women (Masson et al., 2015, 2016). Our data provide insight into the mechanisms by which aSTIs increase HIV risk in men, many of which overlap with known mechanisms in women. The association between inflammation and impaired barrier integrity has been shown to be linked to the FGT (Birse et al., 2016; Berard et al., 2018). Furthermore, asymptomatic infections of CT and NG were shown to dysregulate the CD4+ /CD8+ balance in anogenital epithelia resident T cells. This STI-induced T-cell dysfunction resulted in a significant increase in the expression of immune activation, T-cell exhaustion, and senescence markers in CD8+ T cells (Vieira et al., 2017). Our data here show a shift in the abundance of proteins associated with immune regulation toward an inflammatory response. We also observed enrichment of GO terms associated with myeloid activation, with neutrophil degranulation being enriched in aSTI proteins. Proteins upregulated in aSTI samples had an enriched Interleukin-12-mediated signaling pathway. The expression of interleukin 12 has been shown to increase during CT infection (Hook et al., 2005; Wang et al., 2005) in the FGT and *in vitro* infection studies. Here, we show that in a cis male cohort, where *Chlamydia trichomonas* was the most abundant STI, there was a similar impact on epithelial tissue. We regard this as an important finding, since it extends our previous work (Gray et al., 2019) that CT infection in the male urethra, a mucosal surface, has an impact on the underlying inner foreskin. It possibly may be due to minimal urethral discharge not visible to the naked eye and thereby making its way to the foreskin. Further studies would be needed to verify this.

Interleukin 12 induces differentiation of Th1 cells *via* several steps, including myeloid cell-leukocyte interactions (Bottomly, 1988; Hsieh et al., 1993). Myeloid-derived dendritic cells, including Langerhans cells, stimulate naïve CD4 T cells to mature into Th1 cells which express high levels of the receptor CCR5 (Abbas et al., 1996; Loetscher et al., 1998), potentially rendering them more susceptible to HIV-1 infection due to the HIV-1 co-receptor expression (Bleul et al., 1997; Kawamura et al., 2003; McClure et al., 2005; McKinnon and Kaul, 2012; Prodger et al., 2012; Lemos et al., 2014). We have previously shown that there was an increased presence of CD4+ CCR5+ cells and the dendritic/macrophage-like Langerhans cells in men infected with CT (Gray et al., 2019), and our proteomic data lead to more insight into immune events giving rise to this finding.

The foreskin epithelial proteome has not previously been characterized to our knowledge but is likely an important mediator of susceptibility or protection from HIV, given that circumcision greatly reduces men’s risk of HIV acquisition (Auvert et al., 2005; Bailey et al., 2007; Gray et al., 2007). Our study, however, does have some limitations. The number of aSTIs in our study is small, and a larger cohort would have allowed us to stratify by the type of aSTI and to know whether other STIs, apart from CT, show similar regulation of the epithelial proteome. We also know that the penile microbiome may play an important role in susceptibility to HIV infection (Liu et al., 2017), and future studies investigating the interplay between urethral microbial dysbiosis and epithelial inflammation would provide additional insight. We did find the presence of bacterial proteins that would suggest this link; however, this topic was not further investigated in this study, as we focused on human protein interactions. It has been established that inflammation at other surfaces weakens the epithelial barrier integrity leading to increased leakiness (O’Hara and Buret, 2008; Rao, 2008). The type of inflammation and mechanisms leading to weakened barrier integrity are diverse (Wu et al., 1998; Rao et al., 2002; Bruewer et al., 2003; Gonzalez-Mariscal et al., 2009). At a molecular level, epithelial barrier integrity is controlled by cell adhesion structures, namely, tight junctions, adherens junctions, desmosomes, and gap junctions (Röper and Brown, 2003; Niessen, 2007; Chiba et al., 2008; Hartsock and Nelson, 2008). The apical tight junctions and adherens junctions are integral to the formation and maintenance of epithelial barrier function (Tsukita et al., 2001; Hartsock and Nelson, 2008). We hypothesize based on our data described here that inflammation-induced dysfunction induced by aSTIs possibly weakens the epithelial barrier function, probably by downregulating the expression of homotypic cell-to-cell adhesion. This aSTI-associated decreased barrier function could be a mechanism by which subsequent STI infections, such as HIV, gain easier entry into the mucosa of the MGT. We have seen the increased expression of HIV co-factors involved in infection and immune evasion in the foreskin of men with aSTI. This provides evidence for the syndromic treatment of aSTI to prevent HIV infection. However, previous clinical studies that aimed to reduce the risk of HIV acquisition and transmission by treating STIs had variable success at reducing HIV incidence; this was ascribed to confounders, such as reinfection of STIs by untreated partners, and weak exposure contact (Stillwaggon and Sawers, 2015). In summary, we developed a novel approach to characterizing the foreskin proteome and applied it to successfully distinguish men with and without aSTI. Furthermore, we identify possible mechanisms by which aSTIs and inflammation may possibly increase the risk for HIV acquisition.

## Data availability statement

The datasets presented in this study can be found in online repositories. The names of the repository/repositories and accession number(s) can be found below: http://www.proteomexchange.org/, PXD032798.

## Ethics statement

The studies involving human participants were reviewed and approved by the University of Cape Town HREC. Written informed consent to participate in this study was given by adult participants and in the case of minors less than 18 years old was provided by the participants legal guardian/next of kin.

## Author contributions

NC-M, MP, CG, JB, NM, and L-GB contributed to conception and design of the study. NC-M, BC, SG, ZG, and JB designed and conducted the LC-MS analysis. NC-M, MP, SD, AC, and GB designed and performed the bioinformatic analysis. MP organized the metanovo database. SD and NC-M performed the statistical analysis. NC-M wrote the first draft of the manuscript. SD, HJ, CG, and MP wrote sections of the manuscript. DW, HJ, AO, RH, LM, J-AP, DL, and L-GB contributed to the design, recruitment, and sample collection of the cohort. BN conducted the sample preparation and flow cytometry analysis. All authors contributed to manuscript revision, read, and approved the submitted version.

## Footnotes

1
https://anaconda.org/bioconda/metanovo

2
http://geneontology.org/

## Supplementary material

Supplementary Figure 1 LC-MS^2^ of peptides isolated from a foreskin protein lysate digestion analysis prepared using two digestion methods: (i) IS digestion with subsequent methanol/chloroform extraction of peptides (filter aided sample preparation, FASP) or (ii) protein lysate digestion analysis prepared using IS digestion after protein precipitation using acetone. Top panel shows the ion chromatogram of the MS1, middle panel the total ion current (TIC) of the MS^2^, and the consecutive MS/MS spectrum.

Supplementary Figure 2 GOBubble facet plot showing bubbles representing all gene ontology terms enriched in the three gene ontology categories (biological process (green), cellular component (red), and MF (blue). The z-score depicting the fold changes is presented on the x-axis. The negative logarithm of the adjusted *p*-value is shown on the Y-axis. Functions with an adjusted *p*-value of 0.01 or less are plotted, and only those with a minimum z-score of ∓ 2 are taken. The size of the bubble is proportional to the number of proteins eliciting the function.

Supplementary Datasheet 1 An Excel spreadsheet with a List of proteins identified that were significantly differentially expressed between aSTI proteomes and age-matched controls.

Supplementary Datasheet 2 Negative FC GOs: List of enriched gene ontologies from differentially expressed proteins higher in aSTI- foreskins. Positive FC GOs: List of enriched gene ontologies from differentially expressed proteins higher in aSTI + foreskins. Selected GOs: List of enriched gene ontologies from differentially expressed proteins from aSTI foreskins (in red) and age-matched controls (blue).

## References

[B1] Abbas A. K. Murphy K. M. Sher A. (1996). Functional diversity of helper T lymphocytes. *Nature* 383 787–793. 10.1038/383787a0 8893001

[B2] Agostini L. Martinon F. Burns K. McDermott M. F. Hawkins P. N. Tschopp J. (2004). NALP3 forms an IL-1beta-processing inflammasome with increased activity in Muckle-Wells autoinflammatory disorder. *Immunity* 20 319–325. 10.1016/S1074-7613(04)00046-9 15030775

[B3] Alexa A. Rahnenführer J. (2020). *Gene Set Enrichment Analysis With TopGO.* Available Online at: https://bioconductor.org/packages/release/bioc/html/topGO.html

[B4] Alisoltani A. Manhanzva M. T. Potgieter M. Balle C. Bell L. Ross E. (2020). Microbial function and genital inflammation in young South African women at high risk of HIV infection. *Microbiome* 8 1–21. 10.1186/s40168-020-00932-8 33220709 PMC7679981

[B5] Anzala A. O. Simonsen J. N. Kimani J. Ball T. B. Nagelkerke N. J. Rutherford J. (2000). Acute sexually transmitted infections increase human immunodeficiency virus type 1 plasma viremia, increase plasma type 2 cytokines, and decrease CD4 cell counts. *J. Infect. Dis.* 182 459–466. 10.1086/315733 10915076

[B6] Arnold K. B. Burgener A. Birse K. Romas L. Dunphy L. J. Shahabi K. (2016). Increased levels of inflammatory cytokines in the female reproductive tract are associated with altered expression of proteases, mucosal barrier proteins, and an influx of HIV-susceptible target cells. *Mucosal Immunol.* 9 194–205. 10.1038/mi.2015.51 26104913

[B7] Auvert B. Taljaard D. Lagarde E. Sobngwi-Tambekou J. Sitta R. Puren A. (2005). Randomized, controlled intervention trial of male circumcision for reduction of HIV infection risk: The ANRS 1265 Trial. *PLoS Med.* 2:e298. 10.1371/journal.pmed.0020298 16231970 PMC1262556

[B8] Bailey R. C. Moses S. Parker C. B. Agot K. Maclean I. Krieger J. N. (2007). Male circumcision for HIV prevention in young men in Kisumu, Kenya: A randomised controlled trial. *Lancet* 369 643–656. 10.1016/S0140-6736(07)60312-2 17321310

[B9] Berard A. R. Perner M. Mutch S. Farr Zuend C. McQueen P. Burgener A. D. (2018). Understanding mucosal and microbial functionality of the female reproductive tract by metaproteomics: Implications for HIV transmission. *Am. J. Reprod. Immunol.* 80:e12977. 10.1111/aji.12977 29790240

[B10] Birse K. D. Romas L. M. Guthrie B. L. Nilsson P. Bosire R. Kiarie J. (2016). Genital injury signatures and microbiome alterations associated with depot medroxyprogesterone acetate usage and intravaginal drying practices. *J. Infect. Dis.* 215:jiw590. 10.1093/infdis/jiw590 28011908 PMC5388302

[B11] Bleul C. C. Wu L. Hoxie J. A. Springer T. A. Mackay C. R. (1997). The HIV coreceptors CXCR4 and CCR5 are differentially expressed and regulated on human T lymphocytes. *Proc. Natl. Acad. Sci. U.S.A.* 94 1925–1930. 10.1073/pnas.94.5.1925 9050881 PMC20019

[B12] Bogaerts J. Ahmed J. Akhter N. Begum N. Van Ranst M. Verhaegen J. (1999). Sexually transmitted infections in a basic healthcare clinic in Dhaka, Bangladesh: Syndromic management for cervicitis is not justified. *Sex. Transm. Infect.* 75 437–438. 10.1136/sti.75.6.437 10754954 PMC1758253

[B13] Boily M. C. Anderson R. M. (1996). Human immunodeficiency virus transmission and the role of other sexually transmitted diseases: Measures of association and study design. *Sex. Transm. Dis.* 23 312–332. 10.1097/00007435-199607000-00012 8836026

[B14] Bottomly K. (1988). A functional dichotomy in CD4+ T lymphocytes. *Immunol. Today* 9 268–274. 10.1016/0167-5699(88)91308-4 2908229

[B15] Brès V. Gomes N. Pickle L. Jones K. A. (2005). A human splicing factor, SKIP, associates with P-TEFb and enhances transcription elongation by HIV-1 Tat. *Genes Dev.* 19 1211–1226. 10.1101/gad.1291705 15905409 PMC1132007

[B16] Bruewer M. Luegering A. Kucharzik T. Parkos C. A. Madara J. L. Hopkins A. M. (2003). Proinflammatory cytokines disrupt epithelial barrier function by apoptosis-independent mechanisms. *J. Immunol.* 171 6164–6172. 10.4049/jimmunol.171.11.6164 14634132

[B17] Buckner L. R. Amedee A. M. Albritton H. L. Kozlowski P. A. Lacour N. McGowin C. L. (2016). Chlamydia trachomatis infection of endocervical epithelial cells enhances early HIV transmission events. *PLoS One* 11:e0146663. 10.1371/journal.pone.0146663 26730599 PMC4701475

[B18] Chiba H. Osanai M. Murata M. Kojima T. Sawada N. (2008). Transmembrane proteins of tight junctions. *Biochim. Biophys. Acta* 1778 588–600. 10.1016/j.bbamem.2007.08.017 17916321

[B19] Choi M. Chang C. Y. Clough T. Broudy D. Killeen T. MacLean B. (2014). MSstats: An R package for statistical analysis of quantitative mass spectrometry-based proteomic experiments. *Bioinformatics* 30 2524–2526. 10.1093/bioinformatics/btu305 24794931

[B20] Cox J. Mann M. (2008). MaxQuant enables high peptide identification rates, individualized p.p.b.-range mass accuracies and proteome-wide protein quantification. *Nat. Biotechnol.* 26 1367–1372. 10.1038/nbt.1511 19029910

[B21] Dinh M. H. McRaven M. D. Kelley Z. Penugonda S. Hope T. J. (2010). Keratinization of the adult male foreskin and implications for male circumcision. *AIDS* 24 899–906. 10.1097/QAD.0b013e3283367779 20098294 PMC2951978

[B22] Dixon P. (2003). VEGAN, a package of R functions for community ecology. *J. Veg. Sci.* 14 927–930. 10.1111/j.1654-1103.2003.tb02228.x

[B23] Fernandes-Alnemri T. Wu J. Yu J. W. Datta P. Miller B. Jankowski W. (2007). The pyroptosome: A supramolecular assembly of ASC dimers mediating inflammatory cell death via caspase-1 activation. *Cell Death Differ.* 14 1590–1604. 10.1038/sj.cdd.4402194 17599095 PMC3345951

[B24] Ganor Y. Zhou Z. Tudor D. Schmitt A. Vacher-Lavenu M. C. Gibault L. (2010). Within 1 h, HIV-1 uses viral synapses to enter efficiently the inner, but not outer, foreskin mucosa and engages Langerhans-T cell conjugates. *Mucosal Immunol.* 3 506–522. 10.1038/mi.2010.32 20571487

[B25] Glynn J. R. Biraro S. Weiss H. A. (2009). Herpes simplex virus type 2: A key role in HIV incidence. *AIDS* 23 1595–1598. 10.1097/QAD.0b013e32832e15e8 19512858

[B26] Gonzalez-Mariscal L. Garay E. Lechuga S. (2009). Virus interaction with the apical junctional complex. *Front. Biosci.* 14 731–768. 10.2741/3276 19273098

[B27] Gray C. M. O’Hagan K. L. Lorenzo-Redondo R. Olivier A. J. Amu S. Chigorimbo-Murefu N. (2019). Impact of chemokine C–C ligand 27, foreskin anatomy and sexually transmitted infections on HIV-1 target cell availability in adolescent South African males. *Mucosal Immunol.* 13 118–127. 10.1038/s41385-019-0209-6 31619762 PMC6914668

[B28] Gray R. H. Kigozi G. Serwadda D. Makumbi F. Watya S. Nalugoda F. (2007). Male circumcision for HIV prevention in men in Rakai, Uganda: A randomised trial. *Lancet* 369 657–666. 10.1016/S0140-6736(07)60313-4 17321311

[B29] Hanash S. (2003). Disease proteomics. *Nature* 422 226–232. 10.1038/nature01514 12634796

[B30] Hao Q. Jiao S. Shi Z. Li C. Meng X. Zhang Z. (2015). A non-canonical role of the p97 complex in RIG -I antiviral signaling. *EMBO J.* 34 2903–2920. 10.15252/embj.201591888 26471729 PMC4687688

[B31] Harris D. Engelman A. (2000). Both the structure and DNA binding function of the barrier-to-autointegration factor contribute to reconstitution of HIV type 1 integration in vitro. *J. Biol. Chem.* 275 39671–39677. 10.1074/jbc.M002626200 11005805

[B32] Hartsock A. Nelson W. J. (2008). Adherens and tight junctions: Structure, function and connections to the actin cytoskeleton. *Biochim. Biophys. Acta* 1778 660–669. 10.1016/j.bbamem.2007.07.012 17854762 PMC2682436

[B33] Hirbod T. Bailey R. C. Agot K. Moses S. Ndinya-Achola J. Murugu R. (2010). Abundant expression of HIV target cells and C-type lectin receptors in the foreskin tissue of young Kenyan men. *Am. J. Pathol.* 176 2798–2805. 10.2353/ajpath.2010.090926 20395432 PMC2877841

[B34] Hladik F. McElrath M. J. (2008). Setting the stage: Host invasion by HIV. *Nat. Rev. Immunol.* 8 447–457. 10.1038/nri2302 18469831 PMC2587276

[B35] Ho J. L. He S. Hu A. Geng J. Basile F. G. Almeida M. G. (1995). Neutrophils from human immunodeficiency virus (HIV)-seronegative donors induce HIV replication from HIV-infected patients’ mononuclear cells and cell lines: An in vitro model of HIV transmission facilitated by Chlamydia trachomatis. *J. Exp. Med.* 181 1493–1505. 10.1084/jem.181.4.1493 7699332 PMC2191973

[B36] Hook C. E. Matyszak M. K. Gaston J. S. H. (2005). Infection of epithelial and dendritic cells by *Chlamydia trachomatis* results in IL-18 and IL-12 production, leading to interferon-Î^3^ production by human natural killer cells. *FEMS Immunol. Med. Microbiol.* 45 113–120. 10.1016/j.femsim.2005.02.010 16051062

[B37] Hsieh C. S. Macatonia S. E. Tripp C. S. Wolf S. F. O’Garra A. Murphy K. M. (1993). Development of TH1 CD4+ T cells through IL-12 produced by Listeria-induced macrophages. *Science* 260 547–549. 10.1126/science.8097338 8097338

[B38] Kawamura T. Gulden F. O. Sugaya M. McNamara D. T. Borris D. L. Lederman M. M. (2003). R5 HIV productively infects langerhans cells, and infection levels are regulated by compound CCR5 polymorphisms. *Proc. Natl. Acad. Sci. U.S.A.* 100 8401–8406. 10.1073/pnas.1432450100 12815099 PMC166241

[B39] Kigozi G. Wawer M. Ssettuba A. Kagaayi J. Nalugoda F. Watya S. (2009). Foreskin surface area and HIV acquisition in Rakai, Uganda (size matters). *AIDS* 23 2209–2213. 10.1097/QAD.0b013e328330eda8 19770623 PMC3125976

[B40] Kohl K. S. Markowitz L. E. Koumans E. H. (2003). Developments in the screening for Chlamydia trachomatis: A review. *Obstet. Gynecol. Clin. North Am.* 30 637–658. 10.1016/S0889-8545(03)00076-7 14719842

[B41] LeFevre M. L. U.S. Preventive Services Task Force (2014). Screening for chlamydia and gonorrhea: U.S. Preventive services task force recommendation statement. *Ann. Intern. Med.* 161 902–910. 10.7326/M14-1981 25243785

[B42] Lemos M. P. Lama J. R. Karuna S. T. Fong Y. Montano S. M. Ganoza C. (2014). The inner foreskin of healthy males at risk of HIV infection harbors epithelial CD4+ CCR5+ cells and has features of an inflamed epidermal barrier. *PLoS One* 9:e108954. 10.1371/journal.pone.0108954 25268493 PMC4182607

[B43] Levesque K. Zhao Y.-S. Cohen E. A. (2003). Vpu exerts a positive effect on HIV-1 infectivity by down-modulating CD4 receptor molecules at the surface of HIV-1-producing Cells . *J. Biol. Chem.* 278 28346–28353. 10.1074/jbc.M300327200 12746459

[B44] Levine W. C. Pope V. Bhoomkar A. Tambe P. Lewis J. S. Zaidi A. A. (1998). Increase in endocervical CD4 lymphocytes among women with nonulcerative sexually transmitted diseases. *J. Infect. Dis.* 177 167–174. 10.1086/513820 9419184

[B45] Li Y. P. (1997). Protein B23 is an important human factor for the nucleolar localization of the human immunodeficiency virus protein Tat. *J. Virol.* 71 4098–4102. 10.1128/jvi.71.5.4098-4102.1997 9094689 PMC191564

[B46] Liu C. M. Hungate B. A. Tobian A. A. Serwadda D. Ravel J. Lester R. (2013). Male circumcision significantly reduces prevalence and load of genital anaerobic bacteria. *MBio* 4:e00076. 10.1128/mBio.00076-13 23592260 PMC3634604

[B47] Liu C. M. Prodger J. L. Tobian A. A. R. Abraham A. G. Kigozi G. Hungate B. A. (2017). Penile anaerobic dysbiosis as a risk factor for HIV infection. *MBio* 8:e00996–17. 10.1128/mBio.00996-17 28743816 PMC5527312

[B48] Loetscher P. Uguccioni M. Bordoli L. Baggiolini M. Moser B. Chizzolini C. (1998). CCR5 is characteristic of Th1 lymphocytes [6]. *Nature* 391 344–345. 10.1038/34814 9450746

[B49] Masson L. Arnold K. B. Little F. Mlisana K. Lewis D. A. Mkhize N. (2016). Inflammatory cytokine biomarkers to identify women with asymptomatic sexually transmitted infections and bacterial vaginosis who are at high risk of HIV infection. *Sex. Transm. Infect.* 92 186–193. 10.1136/sextrans-2015-052072 26511781 PMC6801014

[B50] Masson L. Barnabas S. Deese J. Lennard K. Dabee S. Gamieldien H. (2019). Inflammatory cytokine biomarkers of asymptomatic sexually transmitted infections and vaginal dysbiosis: A multicentre validation study. *Sex. Transm. Infect.* 95 5–12. 10.1136/sextrans-2017-053506 30018088

[B51] Masson L. Passmore J. A. Liebenberg L. J. Werner L. Baxter C. Arnold K. B. (2015). Genital inflammation and the risk of HIV acquisition in women. *Clin. Infect. Dis.* 61 260–269. 10.1093/cid/civ298 25900168 PMC4565995

[B52] McClure C. P. Tighe P. J. Robins R. A. Bansal D. Bowman C. A. Kingston M. (2005). HIV coreceptor and chemokine ligand gene expression in the male urethra and female cervix. *AIDS* 19 1257–1265. 10.1097/01.aids.0000180096.50393.96 16052080

[B53] McEver R. P. Zhu C. (2010). Rolling cell adhesion. *Annu. Rev. Cell Dev. Biol.* 26 363–396. 10.1146/annurev.cellbio.042308.113238 19575676 PMC3557855

[B54] McKinnon L. R. Kaul R. (2012). Quality and quantity: Mucosal CD4+ T cells and HIV susceptibility. *Curr. Opin. HIV AIDS* 7 195–202. 10.1097/COH.0b013e3283504941 22314505

[B55] Mlisana K. Naicker N. Werner L. Roberts L. van Loggerenberg F. Baxter C. (2012). Symptomatic vaginal discharge is a poor predictor of sexually transmitted infections and genital tract inflammation in high-risk women in south africa. *J. Infect. Dis.* 206 6–14. 10.1093/infdis/jis298 22517910 PMC3490689

[B56] Nel A. J. Garnett S. Blackburn J. M. Soares N. C. (2015). Comparative reevaluation of FASP and enhanced FASP methods by LC-MS/MS. *J. Proteome Res.* 14 1637–1642. 10.1021/pr501266c 25619111

[B57] Niessen C. M. (2007). Tight junctions/adherens junctions: Basic structure and function. *J. Invest. Dermatol.* 127 2525–2532. 10.1038/sj.jid.5700865 17934504

[B58] O’Hara J. R. Buret A. G. (2008). Mechanisms of intestinal tight junctional disruption during infection. *Front. Biosci.* 13 7008–7021. 10.2741/3206 18508712

[B59] Potgieter M. G. Jm Nel A. Fortuin S. Garnett S. Wendoh J. M. Tabb D. L. (2019). MetaNovo: A probabilistic approach to peptide and polymorphism discovery in complex metaproteomic datasets. *bioRxiv* [Preprint]. 10.1101/605550 PMC10310047 37327214

[B60] Prodger J. L. Gray R. Kigozi G. Nalugoda F. Galiwango R. Hirbod T. (2012). Foreskin T-cell subsets differ substantially from blood with respect to HIV co-receptor expression, inflammatory profile, and memory status. *Mucosal Immunol.* 5 121–128. 10.1038/mi.2011.56 22089029 PMC3288185

[B61] QuickGO (2022). *Term GO:0034109.* Available Online at: https://www.ebi.ac.uk/QuickGO/term/GO:0034109 (accessed September 16, 2022).

[B62] Ramjee G. Sartorius B. Morris N. Wand H. Reddy T. Yssel J. D. (2019). A decade of sustained geographic spread of HIV infections among women in Durban, South Africa. *BMC Infect. Dis.* 19:500. 10.1186/s12879-019-4080-6 31174475 PMC6555962

[B63] Rao R. (2008). Oxidative stress-induced disruption of epithelial and endothelial tight junctions. *Front. Biosci.* 13 7210–7226. 10.2741/3223 18508729 PMC6261932

[B64] Rao R. K. Basuroy S. Rao V. U. Karnaky K. J. Gupta A. (2002). Tyrosine phosphorylation and dissociation of occludin-ZO-1 and E-cadherin-beta-catenin complexes from the cytoskeleton by oxidative stress. *Biochem. J.* 368 471–481. 10.1042/bj20011804 12169098 PMC1222996

[B65] Röper K. Brown N. H. (2003). Maintaining epithelial integrity: A function for gigantic spectraplakin isoforms in adherens junctions. *J. Cell Biol.* 162 1305–1315. 10.1083/jcb.200307089 14517208 PMC2173965

[B66] Shevchenko A. Tomas H. Havlis J. Olsen J. V. Mann M. (2006). In-gel digestion for mass spectrometric characterization of proteins and proteomes. *Nat. Protoc.* 1 2856–2860. 10.1038/nprot.2006.468 17406544

[B67] Stamm W. E. Handsfield H. H. Rompalo A. M. Ashley R. L. Roberts P. L. Corey L. (1988). The association between genital ulcer disease and acquisition of HIV infection in homosexual men. *JAMA* 260 1429–1433. 10.1001/jama.1988.03410100119036 3404600

[B68] Stillwaggon E. Sawers L. (2015). Rush to judgment: The STI-treatment trials and HIV in sub-Saharan Africa. *J. Int. AIDS Soc.* 18:19844. 10.7448/IAS.18.1.19844 25990095 PMC4438085

[B69] Szklarczyk D. Gable A. L. Lyon D. Junge A. Wyder S. Huerta-Cepas J. (2019). STRING v11: Protein-protein association networks with increased coverage, supporting functional discovery in genome-wide experimental datasets. *Nucleic Acids Res.* 47 D607–D613. 10.1093/nar/gky1131 30476243 PMC6323986

[B70] Tsukita S. Furuse M. Itoh M. (2001). Multifunctional strands in tight junctions. *Nat. Rev. Mol. Cell Biol.* 2 285–293. 10.1038/35067088 11283726

[B71] Vieira V. A. Avelino-Silva V. I. Cerqueira N. B. Costa D. A. Costa P. R. Vasconcelos R. P. (2017). Asymptomatic anorectal Chlamydia trachomatis and *Neisseria gonorrhoeae* infections are associated with systemic CD8+ T-cell activation. *AIDS* 31 2069–2076. 10.1097/QAD.0000000000001580 28692536

[B72] Wang C. Tang J. Crowley-Nowick P. A. Wilson C. M. Kaslow R. A. Geisler W. M. (2005). Interleukin (IL)-2 and IL-12 responses to Chlamydia trachomatis infection in adolescents. *Clin. Exp. Immunol.* 142 548–554. 10.1111/j.1365-2249.2005.02946.x 16297168 PMC1809528

[B73] Wang S. Song R. Wang Z. Jing Z. Wang S. Ma J. (2018). S100A8/A9 in Inflammation. *Front. Immunol.* 9:1298. 10.3389/fimmu.2018.01298 29942307 PMC6004386

[B74] Weydert C. van Heertum B. Dirix L. De Houwer S. De Wit F. Mast J. (2018). Y-box-binding protein 1 supports the early and late steps of HIV replication. *PLoS One* 13:e0200080. 10.1371/journal.pone.0200080 29995936 PMC6040738

[B75] Wiesenfeld H. C. Hillier S. L. Krohn M. A. Amortegui A. J. Heine R. P. Landers D. V. (2002). Lower genital tract infection and endometritis: Insight into subclinical pelvic inflammatory disease. *Obstet. Gynecol.* 100 456–463. 10.1097/00006250-200209000-00011 12220764

[B76] Wu S. Lim K. C. Huang J. Saidi R. F. Sears C. L. (1998). *Bacteroides* fragilis enterotoxin cleaves the zonula adherens protein, E-cadherin. *Proc. Natl. Acad. Sci. U.S.A.* 95 14979–14984. 10.1073/pnas.95.25.14979 9844001 PMC24561

