# Supplementary figures and images for: A pilot study to show that asymptomatic sexually transmitted infections alter the foreskin epithelial proteome
Source: Front Microbiol. 2022 Oct 17;13:928317. doi: 10.3389/fmicb.2022.928317 (PMC9618803; doi:10.3389/fmicb.2022.928317)

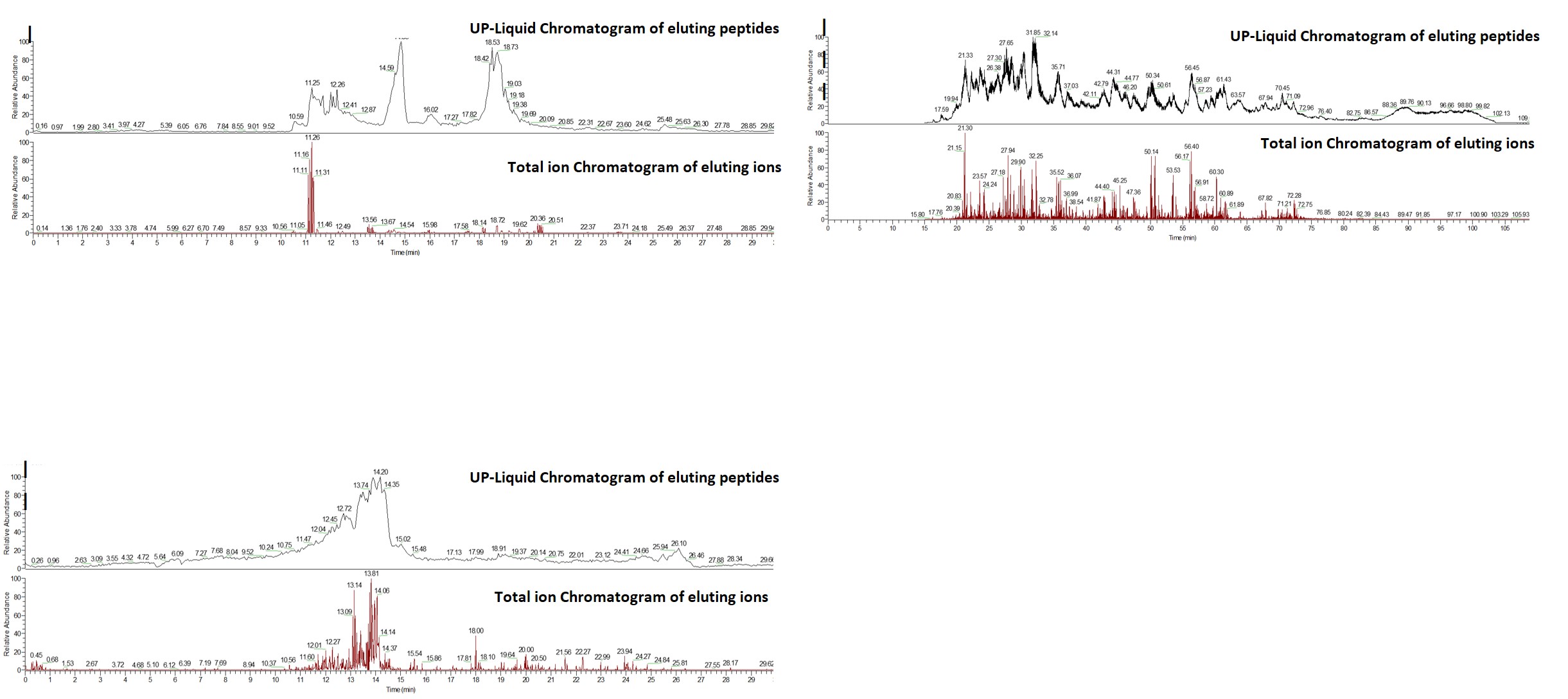

Supplement: Supplementary Figure 1 — LC-MS2 of peptides isolated from a foreskin protein lysate digestion analysis prepared using two digestion methods: (i) IS digestion with subsequent methanol/chloroform extraction of peptides (filter aided sample preparation, FASP) or (ii) protein lysate digestion analysis prepared using IS digestion after protein precipitation using acetone. Top panel shows the ion chromatogram of the MS1, middle panel the total ion current (TIC) of the MS2, and the consecutive MS/MS spectrum. [file Image_1.JPEG]

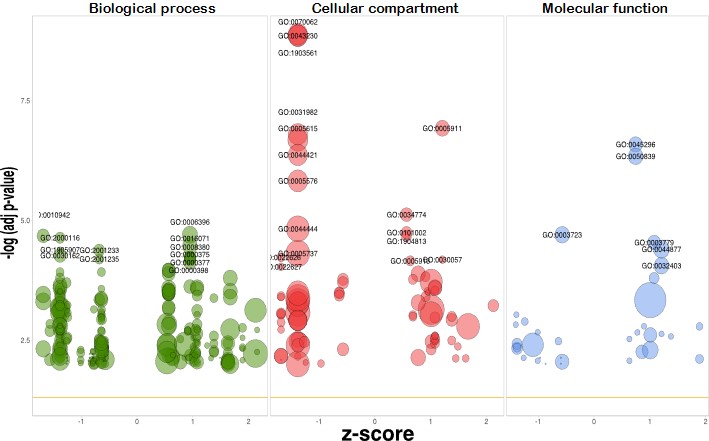

Supplement: Supplementary Figure 2 — GOBubble facet plot showing bubbles representing all gene ontology terms enriched in the three gene ontology categories (biological process (green), cellular component (red), and MF (blue). The z-score depicting the fold changes is presented on the x-axis. The negative logarithm of the adjusted p-value is shown on the Y-axis. Functions with an adjusted p-value of 0.01 or less are plotted, and only those with a minimum z-score of ∓ 2 are taken. The size of the bubble is proportional to the number of proteins eliciting the function. [file Image_2.JPEG]
